# Exosomal hsa_circ_0125310 promotes cell proliferation and fibrosis in diabetic nephropathy via sponging miR‐422a and targeting the IGF1R/p38 axis

**DOI:** 10.1111/jcmm.17065

**Published:** 2021-12-02

**Authors:** Yingchun Zhu, Fangfang Zha, Bo Tang, Ting‐Ting Ji, Xiao‐Ying Li, Linhong Feng, Shou‐Jun Bai

**Affiliations:** ^1^ Department of Nephrology Qingpu Branch of Zhongshan Hospital Fudan University Shanghai China

**Keywords:** diabetic nephropathy, fibrosis, hsa_circ_0125310, MCs, proliferation

## Abstract

Diabetic nephropathy (DN) is still on the rise worldwide, and millions of patients have to be treated through dialysis or transplant because of kidney failure caused by DN. Recent reports have highlighted circRNAs in the treatment of DN. Herein, we aimed to investigate the mechanism by which high glucose‐induced exo‐circ_0125310 promotes diabetic nephropathy progression. circ_0125310 is highly expressed in diabetic nephropathy and exosomes isolated from high glucose‐induced mesangial cells (MCs). High glucose‐induced exosomes promote the proliferation and fibrosis of MCs. However, results showed that the effects of exosomes on MCs can be reversed by the knockdown of circ_0125310. miR‐422a, which targets IGF1R, was the direct target of circ_0125310. circ_0125310 regulated IGF1R/p38 axis by sponging miR‐422a. Exo‐circ_0125310 increased the luciferase activity of the WT‐IGF1R reporter in the dual‐luciferase reporter gene assays and upregulated the expression level of IGF1R and p38. Finally, in vivo research indicated that the overexpression of circ_0125310 promoted the diabetic nephropathy progression. Above results demonstrated that the high glucose‐induced exo‐circ_0125310 promoted cell proliferation and fibrosis in diabetic nephropathy via sponging miR‐422a and targeting the IGF1R/p38 axis.

## 1 INTRODUCTION

Diabetic nephropathy (DN) has been recognized as a complication in patients with diabetes.^1^, ^2^, ^3^ Millions of patients have to betreated through dialysis or transplant because of kidney failure caused by DN.^4^, ^5^, ^6^ Despite the continuous improvement in drugs and technology, DN remains an outstanding problem worldwide. Exploring potential mechanisms and finding effective treatment targets are vital for overcoming this clinical problem.^7^, ^8^ Several studies have demonstrated that exosomes are important communication carriers between cells and can introduce various genetic materials from different cells.^9^, ^10^

Exosomes are a type of microvesicle with a diameter of 40–100 nm and play a significant role in the development of DN.^11^, ^12^ Exosomes are message transmitters that contain DNA, RNA and proteins.^13^ Previous studies have demonstrated that exosomes may act as vital carriers of therapeutic targets in fibrosis.^14^ The downregulation of exosome‐HuR decreases the severity of fibrosis in DN‐associated heart.^15^ Exosomes isolated from mesenchymal stromal cells suppress fibrosis progression in lungs by targeting monocyte phenotypes.^16^ Through in‐depth research, exosomes are recognized as important carriers for circular RNAs (circRNAs), which play vital roles in DN progression.

CircRNAs are noncoding RNA molecules characterized by a covalent closed‐loop free of 3′ and 5′ ends.^17^ Competing endogenous RNAs (ceRNAs) represent a vital communication mechanism between circRNAs and microRNAs (miRNAs). miRNAs, which are small noncoding RNAs with length of 20–25 nucleotides, regulate the expression of downstream targets through post‐transcriptional modulation.^18^, ^19^ In particular, circRNAs serve as molecular sponges and adsorb various miRNAs following the base complementary pairing principle.^20^ circRNAs participate in cell proliferation and tissue fibrosis. The downregulation of circHIPK3 inhibits fibroblast‐to‐myofibroblast transition in pulmonary fibrosis by sponging miR‐338‐3p.^21^ Fibrosis‐associated genes, including CTGF and Col1a2, are upregulated by circ_000203 in cardiac fibroblasts.^22^ circRNAs also play different roles in DN progression. The extracellular matrix of mesangial cells (MCs) is increased by circ_15698 in DN.^23^ circLRP6 enhances high glucose‐associated oxidative stress in MCs.^24^ circ_0080425 acts as a suppressor in DN progression by targeting miR‐24‐3p.^25^ However, the roles of exosome circRNAs in DN progression are largely unclarified.

circ_0125310 is a marker of diabetes complications.^26^ However, the precise role of circ_0125310 is unknown. In this study, circ_0125310 was found to be highly expressed in exosomes isolated from high glucose‐induced MCs. Moreover, exo‐circ_0125310 promoted the proliferation and fibrosis of MCs. Furthermore, miR‐422a was identified as the direct target of circ_0125310 through a ceRNA‐ and Ago2‐dependent manner. Then, exo‐circ_0125310 activated the insulin‐like growth factor I receptor (IGF1R)/p38 axis by sponging miR‐422a. The data from in vivo studies illustrated that the overexpression of circ_0125310 promoted DN progression and regulated miR‐422a/IGF1R/p38 axis in a DN rat model.

## 2 MATERIALS AND METHODS

### 2.1 Tissues

In total, 56 samples of kidney tissues and serum of patients were obtained from Qingpu Branch of Zhongshan Hospital. These samples were all stored at −80°C until use. This study was approved by the Medical Ethics Committee of Qingpu Branch of Zhongshan Hospital Affiliated to Fudan University.

### 2.2 Cell culture

MCs were cultured in an incubator (37 °C, 5% CO_2_) and in RPMI1640 (Gibco, Grand Island, NY, USA) supplemented with 10% FBS (Gibco, Grand Island, NY, USA). MCs were cultured in normal (NC group, 5.5 mM glucose) and high (HG group, 30 mM glucose) glucose.

### 2.3 Flow cytometry analysis

MCs were treated with exosomes or LV/sh‐circ_0125310 for 48 h. Then, transfected MCs were fixed in ethanol (4°C) for 12 h. Prior to testing, MCs were washed thrice with PBS (4°C, Gibco, Grand Island, NY, USA) and stained with prefabricated PI/Triton X‐100 (containing 0.1% Triton X‐100 and 20 µg PI). Then, MCs were incubated for 25 min in dark conditions. Finally, RNase A (0.25 mg) was added to the MCs. Flow cytometry was operated through a standardized process.

### 2.4 Cell proliferation assay

The proliferation of MCs was evaluated through CCK8 and EDU assays. Cells were cultured in RPMI1640 without FBS for 12 hours. For EDU assays, MCs (9 × 10^4^) were plated in 6‐well inserts and treated with 50 μm EdU (Beyotime, China). Then, MCs were incubated for 10 h. Then, MCs were fixed with 4% paraformaldehyde for half an hour at standard temperature (25°C). For CCK8 assays, MCs (5 × 10^4^) were plated in 24‐well inserts and a microplate reader (450 nm) was used to determine the numerical value. Before testing, the MCs were treated with 12 µM CCK8 (Beyotime, China) reagent and transferred to 96‐well plates (100 µl DMEM) after 2 h of incubation at standard temperature (25°C).

### 2.5 Reverse transcription PCR and qTR‐PCR

The total RNA of cells or tissues was extracted using TRIzol Reagent (Invitrogen, CA, USA). The HiScript II Q Select RT Supermix was used for reverse transcription PCR. IGF1R, p38 and GAPDH expression levels were detected using the following primers:

F‐5′‐TCGACATCCGCAACGACTATC‐3′ and

R‐5′‐ CCAGGGCGTAGTTGTAGAAGAG‐3′;

F‐5′‐GCGGAGTAGCTGGTACTGG‐3′ and

R‐5′‐ GCGCGAGTTCTCTGAGACG‐3′; and

F‐5′‐GGAGCGAGATCCCTCCAAAAT‐3′ and

R‐5′‐ GGCTGTTGTCATACTTCTCATGG‐3′.

The SYBR Green Master Mix (Takara, Shiga, Japan) was used to measure the expression level of genes. The fold changes were calculated using the formula 2^−(ΔΔCt)^.

### 2.6 Lentivirus production and cell transfection

LV/sh‐circ_0125310 was established by Shanghai Genechem Co (China). The mimic‐ and inhibitor‐miR‐422a were purchased from RiboBio (Guangzhou, China). The pcDNA3.1 (blank vector) and pcDNA3.1‐IGF1R were purchased from GiKa (Shanghai, China). Lipofectamine 2000 (Invitrogen, CA, USA) was used for transfection in accordance with the manufacturer's instructions.

### 2.7 Dual‐luciferase reporter assay

A segment of the circ_0125310 region contained the binding sites for miR‐422a predicted using the StarBase (http://starbase.sysu.edu.cn). The MUT‐circ_0125310 binding sites were established, and the binding sites for miR‐422a were knocked out. MCs were cotransfected with WT/MUT‐circ_0125310 or mimic/inhibitor‐miR‐422a. Luciferase activity was measured using the Dual‐Luciferase Reporter Assay System (Toyo Ink, Tokyo, Japan).

### 2.8 RNA immunoprecipitation assays

The cell lysate was incubated with RIP immunoprecipitation buffer following introduction. The buffer contained Ago2 antibody (Millipore, Billerica, MA, USA) and magnetic beads conjugated with IgG (NC, Millipore, Billerica, MA, USA). The coprecipitated RNAs were detected using gel electrophoresis and qPCR.

### 2.9 Immunofluorescence

The DN tissues were fixed with precooled paraformaldehyde (4°C). After permeabilization with Triton X‐100 (0.2%) for 12 min, the DN tissues were confined for 2 h. Then, the DN tissues were incubated with the Phospho‐H2AFX‐S139 antibody (1:50, Abcam, Eugene, USA) at 4°C for 12 h. The antibody contained ABclonal and AP0099 (1:100, Abcam, Eugene, USA). Finally, antirabbit IgG and DAPI were used for nuclear staining in the dark.

### 2.10 Western blotting

Each sample was prepared in precooled RIPA buffer (4°C). From total protein, 16–20 mg protein was transferred onto PVDF membranes. The antibodies used in this study included anti‐p‐cadherin (1:1000; Cell Signaling Technology, USA), anti‐ZO‐1 (1:1000; Cell Signaling Technology, USA), anti‐TGFβ1 (1:1000; Cell Signaling Technology, USA), anti‐IGF1R (1:1000; Cell Signaling Technology, USA), anti‐p38 (1:1000; Cell Signaling Technology, USA) and anti‐GAPDH (1:2000; Cell Signaling Technology, USA). The Image J software was used to analyse the grey value of the protein bands. The target protein is divided by GAPDH in NC group, which is normalized as one.

### 2.11 Animal models

The DN rat models were handled in accordance with the literature.^27^ Male rats (8 weeks, the number of every group = 6) were bought from Laboratory Animal Center (Shanghai, China). Rats were raised in SPF laboratory animal room. The DN rat model was induced using 50 mg/kg STZ through the following process. Once a day for five days, the rats received an intraperitoneal injection of STZ, which was dissolved in citrate acid buffer (0.1 M). Then, the successful model was defined as blood glucose ≥16.5 mmol/L. In accordance with the experimental objective, 18 DN rats were divided into three groups: NC (PBS), LV‐NC and LV‐circ_0125310 groups. A total of 150 µl PBS, LV‐NC or LV‐circ_0125310 was injected into the DN rats through the caudal vein. After four weeks, the rats were killed, and the DN tissues were collected. This study was approved by the Medical Ethics Committee of Qingpu Branch of Zhongshan Hospital Affiliated to Fudan University. All animal experiments were based on the Guide for the Care and Use of Laboratory Animals of the National Institutes of Health.

### 2.12 Statistical analysis

Derived values are presented as mean ± SD. Student's t test and one‐way ANOVA by using the GraphPad Prism 5.0 software and SPSS 13.0 were used to perform statistical analyses. *p* values <0.05 were considered statistically significant.

## 3 RESULTS

### 3.1 circ_0125310 was highly expressed in DN and exosomes isolated from high glucose‐induced MCs

**FIGURE 1:**
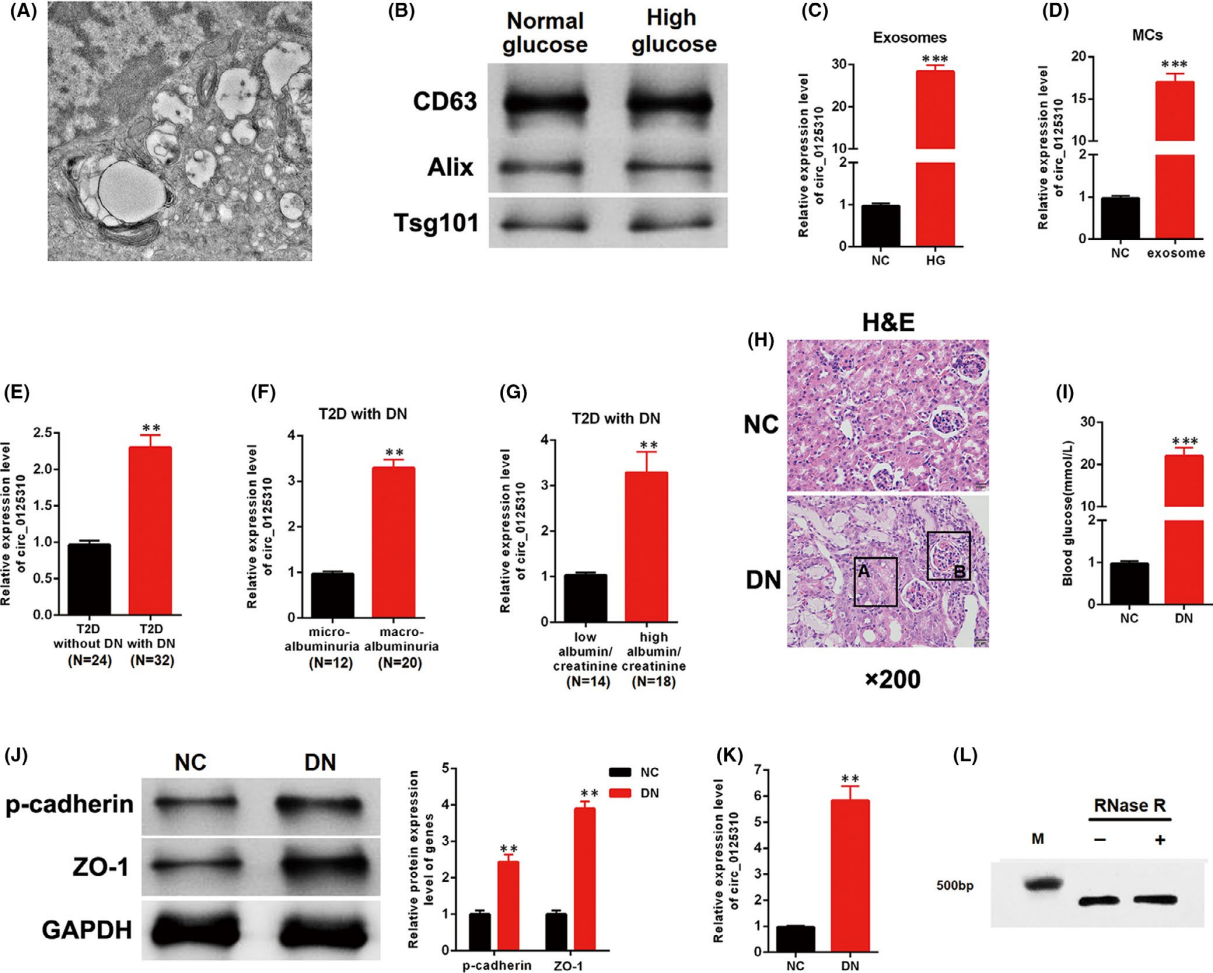
Expression patterns of exo‐circ_0125310 in DN. A, TEM image of exosomes isolated from culture media (scale bar, 100 nm). B, Exosomes were isolated from MCs with or without high glucose. Western blotting of three representative exosome specific markers: CD63, Alix and Tsg101. C, The expression level of circ_0125310 was detected in exosomes from MCs with or without high glucose. D, The expression level of circ_0125310 was detected in MCs treated by exosomes, which were secreted from normal or high glucose. E, The expression level of circ_0125310 was detected in kidney tissues with T2D or without DN (T2D without DN = 24, T2D with DN = 32). F, Expression levels of circ_0125310 was detected in kidney tissues with microalbuminuria or with macroalbuminuria (microalbuminuria group = 12, macroalbuminuria group = 20). G, Based on the average value of eGFR, expression levels of circ_0125310 were detected in kidney tissues with low or high eGFR (low eGFR group = 14, high eGFR group = 18). H, H&E staining results of the DN rat model and negative control. Results indicated that the epithelium cells of tubule were edematous. Glomerular mesangial proliferation is shown in DN (*n* = 6). I, Blood glucose was detected in the DN rat model and negative control (*n* = 6). J, Western blotting of two representative specific markers of renal fibrosis: p‐cadherin and ZO‐1. K, The expression level of circ_0125310 was detected in kidney tissues of NC and DN rat model. L, Validation of circ_0125310 by RNase R treatment and reverse transcription PCR (RT‐PCR) analysis

Exosomes were separately isolated from the culture media of MCs treated with normal or high glucose to detect the expression level of circ_0125310. The TEM image of exosomes is shown in Figure 1A. Three exosome markers, including CD63, Alix and tsg101, were confirmed in the normal and high glucose groups (Figure 1B). The qRT‐PCR results indicated that circ_0125310 was more highly expressed in exosomes from MCs treated with high glucose than in those treated with normal glucose (Figure 1C). Furthermore, exosomes were added to the culture media of MCs. The qRT‐PCR results showed that the expression level of circ_0125310 was upregulated in MCs treated with high glucose‐induced exosomes (Figure 1D). The relationship between circ_0125310 and clinical and pathological data was investigated. circ_0125310 was more highly expressed in T2D with DN than that without DN (Figure 1E). Furthermore, the expression level of circ_0125310 was investigated in T2D with DN. Our finding indicated that circ_0125310 was increased in the macroalbuminuria group (*n *= 20) relative to that in the microalbuminuria group (*n *= 12) (Figure 1F). circ_0125310 was highly expressed in the high albumin/creatinine group (n = 18) relative to that in the low albumin/creatinine group (*n *= 14) (Figure 1G). Furthermore, the DN rat model was established. The H&E staining results showed that swollen epithelial cells (A) and the hyperproliferation of the glomerular mesangium (B) were found in DN rat model (Figure 1H). The expression level of blood glucose was prominently increased in the DN rat model relative to that in the negative control (Figure 1I). Two fibrosis markers, namely p‐cadherin and ZO‐1, were detected in the DN rat model and negative control. The expression levels of fibrosis markers were increased in the DN rat model (Figure 1J). The qRT‐PCR results showed that the expression level of circ_0125310 was increased in the DN rat model (Figure 1K). circ_0125310 was subjected to RNase R treatment to validate the resistance of circ_0125310 to RNase R digestion. Results indicated that circ_0125310 was resistant to RNase R digestion (Figure 1L). Thus, the expression level of circ_0125310 was upregulated in DN and exosomes from high glucose‐induced MCs.

### 3.2 Exo‐circ_0125310 promoted the proliferation and fibrosis of MCs

**FIGURE 2:**
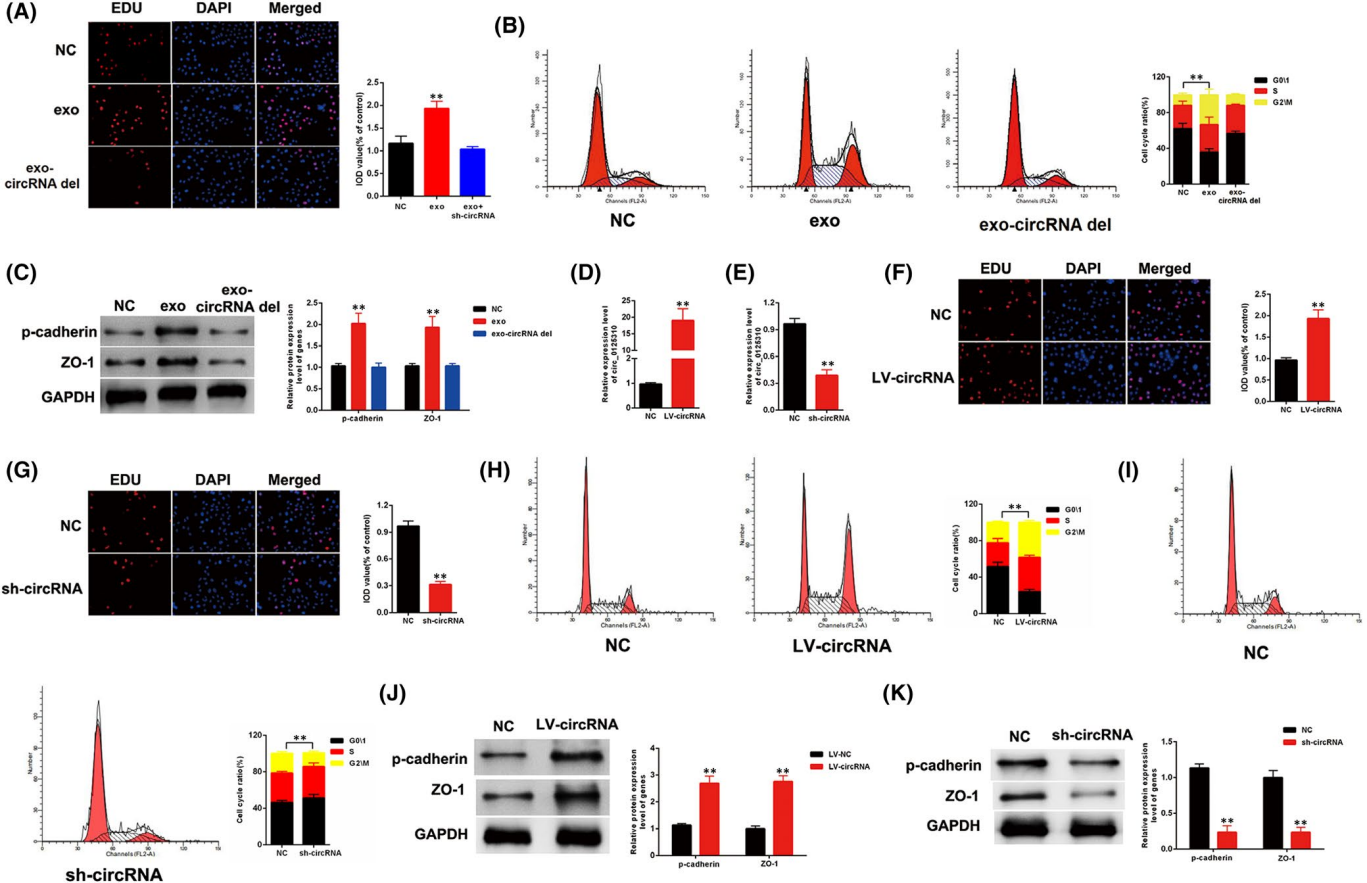
Exo‐circ_0125310 promotes growth and fibrosis of MCs cells. A, Proliferating mesangial cells were labelled with EdU. MCs were treated with NC, exosomes and exosomes‐circRNA del. B, Flow cytometric assay showed that G2/M phase of MCs was increased in MCs treated with exosomes, but this promotion was eliminated when circ_0125310 was knocked down in exosomes. C, Western blotting results showed that the expression level of p‐cadherin and ZO‐1 was upregulated in MCs treated with exosomes, but this promotion was eliminated when circ_0125310 was knocked down in exosomes. D and E, Efficiency of LV‐circ_0125310 and sh‐circ_0125310 were detected by qPCR. F and G, Proliferating mesangial cells were labelled with EdU. MCs were transfected with LV‐circ_0125310 and sh‐circ_0125310. H and I, Flow cytometric assay showed that G2/M phase of MCs was increased in MCs treated with LV‐circ_0125310, but G2/M phase was decreased when circ_0125310 was knocked down in MCs. J and K, LV‐circ_0125310 upregulated the expression level of p‐cadherin and ZO‐1 and sh‐circ_0125310 downregulated the expression level of p‐cadherin and ZO‐1

sh‐circ_0125310 was transfected into MCs treated with high glucose and exosomes, namely, exo‐circ_0125310 del, collected at 48 h post‐transfection to confirm the function of circ_0125310. EDU assays were performed using MCs treated with NC (PBS), exosomes (high glucose) and exo‐circ_0125310 del. Results indicated that exosomes upregulated the growth of MCs and that the upregulation was restored when circ_0125310 was knocked out in MC exosomes (Figure 2A). Flow cytometry analysis was performed. Results indicated that exosomes upregulated the G2/M phase of MCs, and these effects were restored in the exo‐circ_0125310 del group (Figure 2B). Then, the expression levels of p‐cadherin and ZO‐1 were detected in MCs. Results showed that the protein expression levels of p‐cadherin and ZO‐1 were increased in the exosome group relative to that in the NC group, but the downregulation of circ_0125310 in exosomes eliminated this effect (Figure 2C). Furthermore, the function of circ_0125310 was investigated. The efficiency of LV‐circ_0125310 and sh‐circ_0125310 was detected through qPCR. Results showed that the expression level of circ_0125310 was successfully upregulated by LV‐circ_0125310 and downregulated by sh‐circ_0125310 (Figure 2D and 2E). EDU assays indicated that LV‐circ_0125310 and sh‐circ_0125310 enhanced and suppressed, respectively, the growth of MCs (Figure 2F and 2G). Moreover, the G2/M phase of MCs was increased by LV‐circ_0125310 and decreased by sh‐circ_0125310 (Figure 2H and 2I). The protein expression levels of p‐cadherin and ZO‐1 in MCs transfected with LV‐circ_0125310 or sh‐ circ_0125310 were detected by Western blot analysis. Our findings indicated that the expression level of p‐cadherin and ZO‐1 was upregulated by LV‐circ_0125310 but downregulated by sh‐circ_0125310 (Figure 2J and 2K). Thus, the above results indicated that exo‐circ_0125310 promoted the proliferation and fibrosis of MCs.

### 3.3 miR‐422a was the direct target of circ_0125310

**FIGURE 3:**
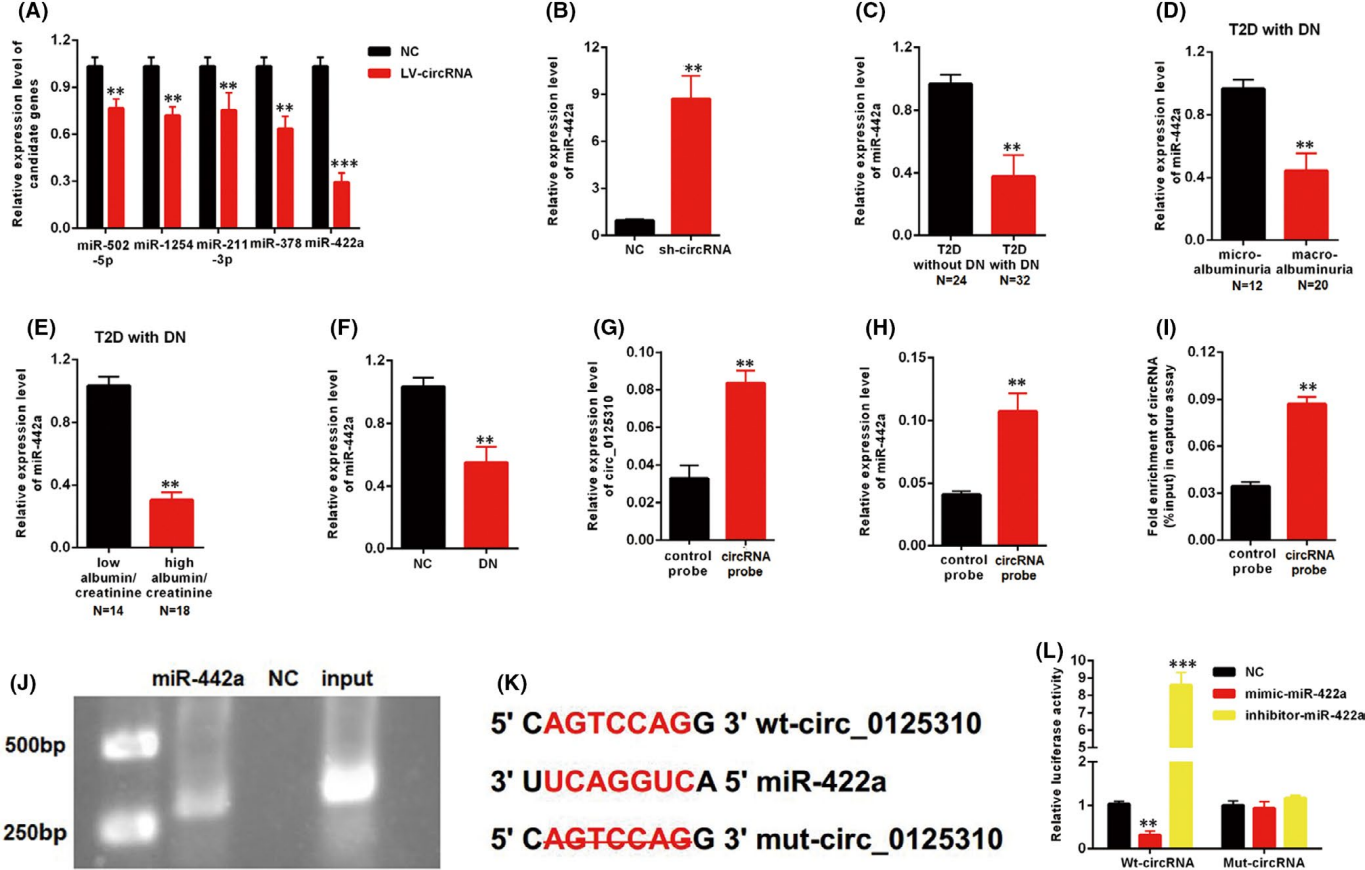
miR‐422a was the directly target of circ_0125310. A, Five miRNAs were screened in MCs transfected with LV‐circ_0125310. B, The expression level of miR‐422a was upregulated in MCs transfected with sh‐circ_0125310. C, The expression level of miR‐422a was decreased in kidney tissues with DN (T2D without DN=24, T2D with DN = 32). D, Expression levels of miR‐422a was downregulated in kidney tissues with macroalbuminuria group (microalbuminuria group = 12, macroalbuminuria group = 20). E, Expression levels of miR‐422a was downregulated in kidney tissues with high eGFR group (low eGFR group = 14, high eGFR group = 18). F, The expression level of miR‐422a was decreased in kidney tissues of the DN rat model. G, circ_0125310 in cell lysis was pulled down and enriched with circ_0125310 specific probe in MCs. H, miR‐422a was pulled down and enriched with circ_0125310 specific probe in MCs. I and J, Biotin‐coupled miR‐422a captured a fold change of circ_0125310 in the complex as compared with biotin‐coupled NC in biotin‐coupled miRNA capture. Then products were detected by agarose gel electrophoresis. K, The direct binding sites between circ_0125310 and miR‐422a were presented. L, Luciferase reporter assay was performed to confirm the direct binding relationship between circ_0125310 and miR‐422a

circMIR (www.bioinf.com.cn) was used to predict the potential miRNA targets of circ_0125310. Five candidates were screened in MCs transfected with LV‐circ_0125310. The qRT‐PCR results indicated the maximum range of discount in the expression level of miR‐422a among other candidates (Figure 3A). Moreover, the downregulation of circ_0125310 upregulated the expression level of miR‐422a (Figure 3B). The relationship between miR‐422a and clinical and pathological data was investigated. The expression level of miR‐422a was downregulated in T2D with DN relative to that in T2D without DN (Figure 3C). Furthermore, the expression level of miR‐422a was investigated in T2D with DN. miR‐422a decreased in the macroalbuminuria group (*n* = 20) relative to that in the microalbuminuria group (*n* = 12) (Figure 3D). miR‐422a was expressed at low levels in the high albumin/creatinine group (*n* = 18) relative to that in the low albumin/creatinine group (*n* = 14) (Figure 3E). In addition, miR‐422a was downregulated in the DN rat model (Figure 3F). circ_0125310 and miR‐422a in cell lysates were enriched by a circ_0125310‐specific probe (Figure 3G–H). Biotin‐coupled miR‐422a captured a fold change of circ_0125310 in the complex compared with the biotin‐coupled NC in MCs. Then, the products were detected through agarose gel electrophoresis (Figure 3I–J). The specific binding site between circ_0125310 and miR‐422a is shown in Figure 3K. As shown in Figure 3L, luciferase activity was decreased by mimic‐miR‐422a but increased by inhibitor‐miR‐422a in the WT‐circ_0125310 reporter. However, the Mut‐circ_0125310 reporter showed no increase or decrease in the luciferase activity of MCs transfected by mimic‐ or inhibitor‐miR‐422a. Therefore, miR‐422a was directly targeted by circ_0125310 through direct targeting and Ago2‐dependent manner.

### 3.4 miR‐422a inhibited the proliferation and fibrosis of MCs by targeting IGF1R

**FIGURE 4:**
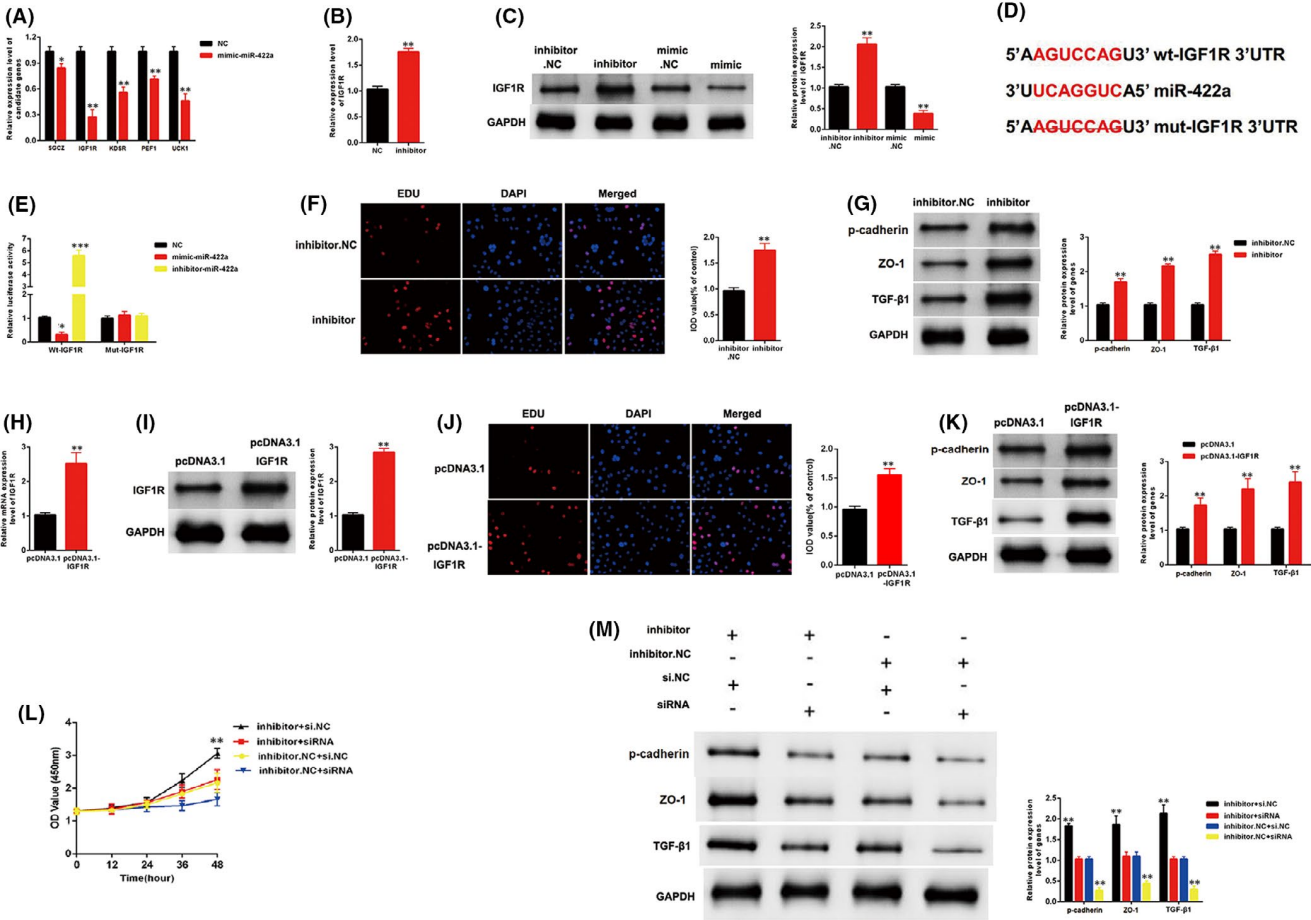
miR‐422a suppressed proliferation and fibrosis of MCs by targeting IGF1R. A, Five genes were screened in MCs transfected with mimic‐miR‐422a. B, The expression level of miR‐422a was upregulated in MCs transfected with inhibitor‐miR‐422a. C, The protein expression level of IGF1R was increased by inhibitor‐miR‐422a and decreased by mimic‐miR‐422a. D, The direct binding sites between miR‐422a and IGF1R were presented. E, Luciferase reporter assay was performed to confirm the direct binding relationship between miR‐422a and IGF1R. F, Proliferating mesangial cells were labelled with EdU. Inhibitor‐miR‐422a enhanced the growth of MCs. G, Inhibitor‐miR‐422a enhanced the expression level of markers of fibrosis: p‐cadherin, ZO‐1 and TGF‐β1. H, and I, Efficiency of pcDNA3.1‐IGF1R were detected by qPCR and western blotting. J, and K, Overexpression of IGF1R enhanced the growth and fibrosis of MCs. L, The CCK8 assays indicated that the growth of MCs was promoted by inhibitor‐miR‐422a, but this promotion was eliminated when IGF1R was knocked down in MCs. M, Western blotting results showed that the expression level of p‐cadherin, ZO‐1 and TGF‐β1 was upregulated in MCs treated with inhibitor‐miR‐422a, but this promotion was restored when IGF1R was downregulated in MCs

Targetscan (http://www.targetscan.org) was used to predict the potential gene targets of miR‐422a. Five candidates were screened in MCs transfected with mimic‐miR‐422a. The qRT‐PCR results indicated a maximum drop in the expression level of IGF1R (Figure 4A). Moreover, the inhibitor‐miR‐422a upregulated the mRNA expression level of IGF1R (Figure 4B). The Western blot analysis results indicated that the protein expression level of IGF1R was decreased by mimic‐miR‐422a and increased by inhibitor‐miR‐422a (Figure 4C). The specific binding site between miR‐422a and IGF1R is shown in Figure 4D. As shown in Figure 4E, luciferase activity was decreased by mimic‐miR‐422a but increased by inhibitor‐miR‐422a in the WT‐IGF1R reporter. However, the Mut‐IGF1R reporter showed no increase or decrease in the luciferase activity of MCs transfected with mimic‐ or inhibitor‐miR‐422a. The inhibitor‐miR‐422a enhanced the growth of MCs and upregulated the expression level of three fibrosis markers, namely, p‐cadherin, ZO‐1 and TGF‐β1 (Figure 4F and 4G). Furthermore, the role of IGF1R was investigated in MCs. The efficiency of pcDNA3.1‐IGF1R was detected by qPCR and Western blot analysis. Results showed that the expression level of IGF1R was successfully overexpressed by pcDNA3.1‐IGF1R (Figure 4H and 4I). IGF1R overexpression enhanced the growth of MCs and upregulated the expression levels of p‐cadherin, ZO‐1 and TGF‐β1 (Figure 4J, 4K). Furthermore, our findings indicated that the si‐IGF1R can restore the upregulation of growth and fibrosis markers in MCs transfected with inhibitor‐miR‐422a (Figure 4L and 4M). Thus, miR‐422a inhibited the proliferation and fibrosis of MCs by targeting IGF1R.

### 3.5 circ_0125310 regulated the IGF1R/p38 axis and promoted the proliferation and fibrosis of MCs cells by acting as a miR‐422a sponge

**FIGURE 5:**
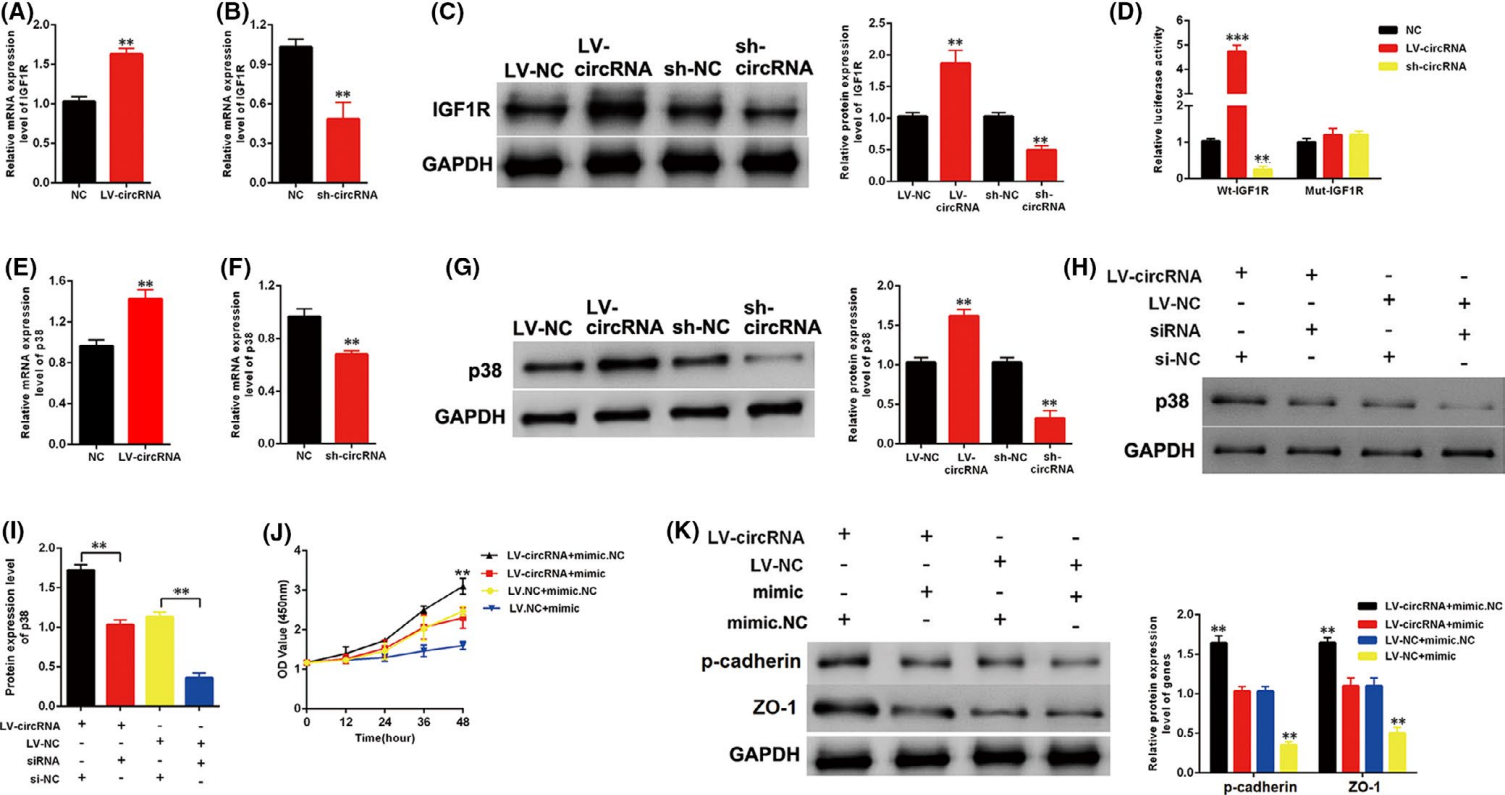
circ_0125310 regulates IGF1R/p38 expression and promoted proliferation and fibrosis of MCs cells by acting as miR‐422a sponge. A, B, and C, The expression level of IGF1R was upregulated by LV‐circ_0125310, but the expression level of IGF1R was downregulated by sh‐circ_0125310. D, The mutant‐type reporter gene (Mut‐IGF1R reporter) was established, which binds sites of miR‐422a and IGF1R was knockout. Luciferase reporter assay was performed to confirm the relationship between circ_0125310 and IGF1R. E, F and G, The expression level of p38 was upregulated by LV‐circ_0125310, but the expression level of p38 was downregulated by sh‐circ_0125310. H and I, Western blotting results showed that the expression level of p38 was upregulated in MCs treated with LV‐circ_0125310, but this promotion was restored when IGF1R was downregulated in MCs. J, The CCK8 assays indicated that the proliferation of MCs was promoted by LV‐circ_0125310, but this promotion was restored when MCs was transfected by mimic‐miR‐422a. K, The protein expression level of p‐cadherin and ZO‐1 was upregulated in MCs treated with LV‐circ_0125310, but this upregulation was restored when MCs were transfected by mimic‐miR‐422a

The relationship between circ_0125310 and IGF1R was investigated. As shown in (Figure 5A–C), the expression level of IGF1R was upregulated by LV‐circ_0125310 but downregulated by sh‐circ_0125310. Furthermore, the Mut‐IGF1R reporter, which bound sites of miR‐422a and IGF1R, was knocked out and established. A luciferase reporter assay was performed using MCs. Luciferase activity was increased by LV‐circ_0125310 but decreased by sh‐circ_0125310 in the WT‐IGF1R reporter. However, the Mut‐IGF1R reporter showed no increase or decrease in the luciferase activity of MCs transfected by LV‐circ_0125310 or sh‐circ_0125310 (Figure 5D). A previous study has demonstrated that the IGF1R/p38 axis is a vital signalling pathway in DN.^28^ The expression level of p38 was detected in MCs transfected with LV‐circ_0125310 or sh‐circ_0125310. The qRT‐PCR and the Western blot analysis results showed that the expression level of p38 was upregulated by LV‐circ_0125310 and downregulated by sh‐circ_0125310 (Figure 5E, 5G). Furthermore, the Western blot analysis indicated that the upregulation of p38 in MCs after transfection with LV‐circ_0125310 can be restored by si‐IGF1R (Figure 5H and 5I). In addition, our findings indicated that mimic‐miR‐422a can restore the upregulation of growth and fibrosis markers in MCs transfected with LV‐circ_0125310 (Figure 5J and 5K). Thus, circ_0125310 upregulated the expression levels of IGF1R and p38 and promoted the proliferation and fibrosis of MCs by targeting miR‐422a.

### 3.6 Exo‐circ_0125310 regulated the miR‐422a/IGF1R/p38 axis

**FIGURE 6:**
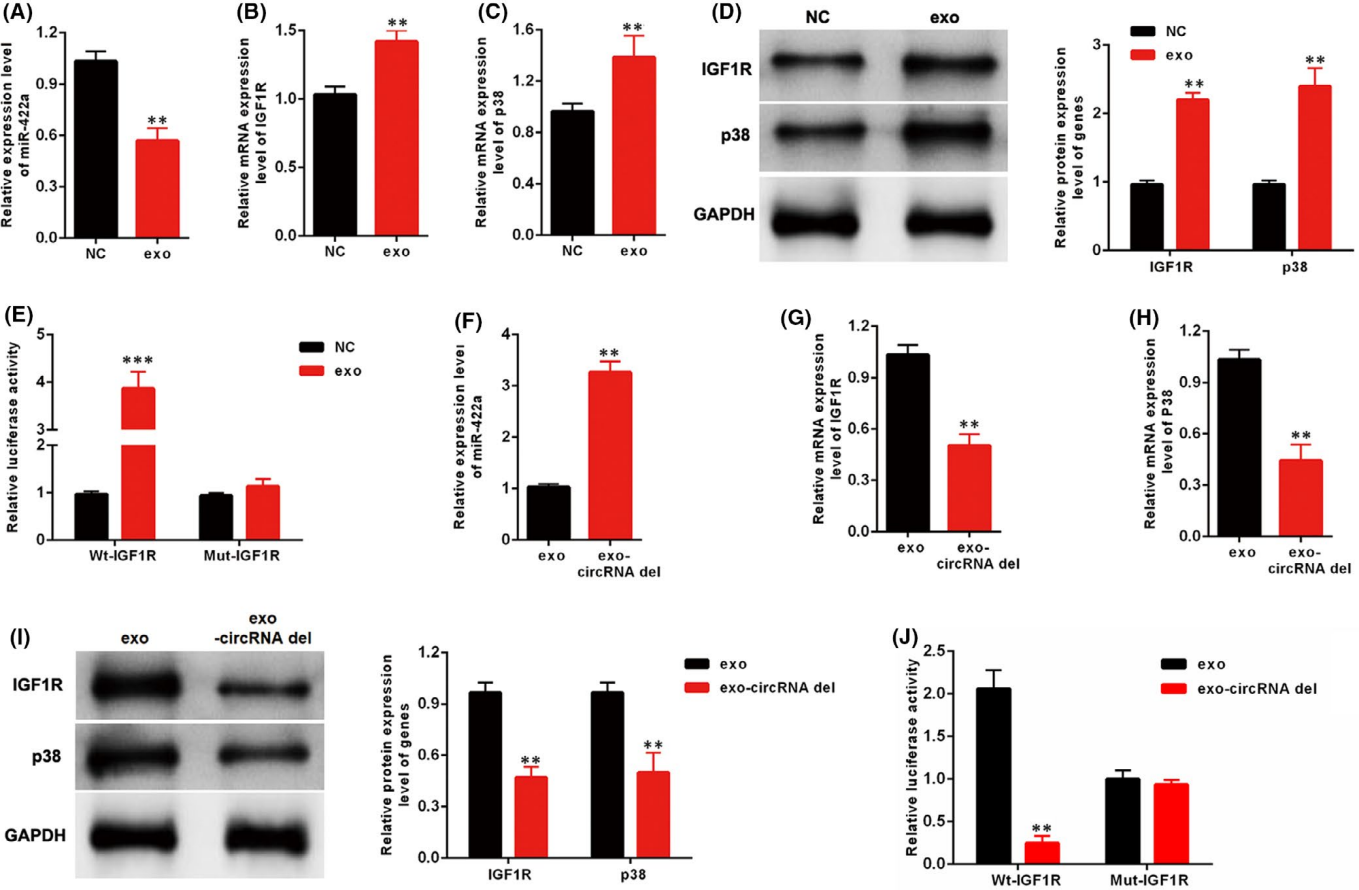
Effects of exo‐circ_0125310 on miR‐422a and IGF1R/p38 axis in MCs cells. A, The expression level of miR‐422a was detected in MCs treated with NC or exosomes. B, C and D, The expression level of IGF1R and p38 was detected in MCs treated with NC or exosomes. E, The Mut‐IGF1R reporter was established, which binds sites of circ_0125310 and IGF1R was knockout. Luciferase reporter assay was performed to confirm the relationship between exosomes and IGF1R. F, The expression level of miR‐422a was detected in MCs treated with exosomes or exo‐circRNA del. G, H, and I, The expression level of IGF1R and p38 was detected in MCs treated with exosomes or exo‐circRNA del. J Luciferase reporter assay was performed to confirm the relationship between exo‐circRNA del and IGF1R

The effects of exosomes on the miR‐422a/IGF1R/p38 axis were explored. After 24h of incubation, the qRT‐PCR results indicated that the expression levels of miR‐422a were downregulated and IGF1R, and p38 were upregulated in MCs treated with exosomes relative to those in the negative control (Figure 6A–C **)**. Moreover, the Western blot analysis results showed that the protein expression levels of IGF1R and p38 were increased in MCs after incubation with exosomes induced by high glucose (Figure 6D). As shown in Figure 6E, luciferase activity was increased by exosomes induced by high glucose in the WT‐IGF1R reporter, but the Mut‐IGF1R reporter (knockout bind sites of miR‐422a and IGF1R) showed no increase or decrease in the luciferase activity of MCs treated with exosomes or NC. Furthermore, MCs were incubated with exosomes or exo‐circ_0125310 del. After 24h of incubation, qRT‐PCR showed that silencing circ_0125310 in exosomes restored the downregulation of miR‐422a in MCs treated by exosomes (Figure 6F). Moreover, qRT‐PCR and Western blot analysis showed that the expression levels of IGF1R and p38 in the exo‐circ_0125310 del group were lower than those in the exosome group (Figure 6G–I). Then, as shown in Figure 6J, luciferase activity was increased by exosomes than in exo‐circ_0125310 del group in the WT‐IGF1R reporter, but the Mut‐IGF1R reporter (knockout bind sites of miR‐422a and IGF1R) showed no increase or decrease in the luciferase activity of MCs treated by exosomes or NC.

### 3.7 Overexpression of circ_0125310 promoted DN progression in vivo

**FIGURE 7:**
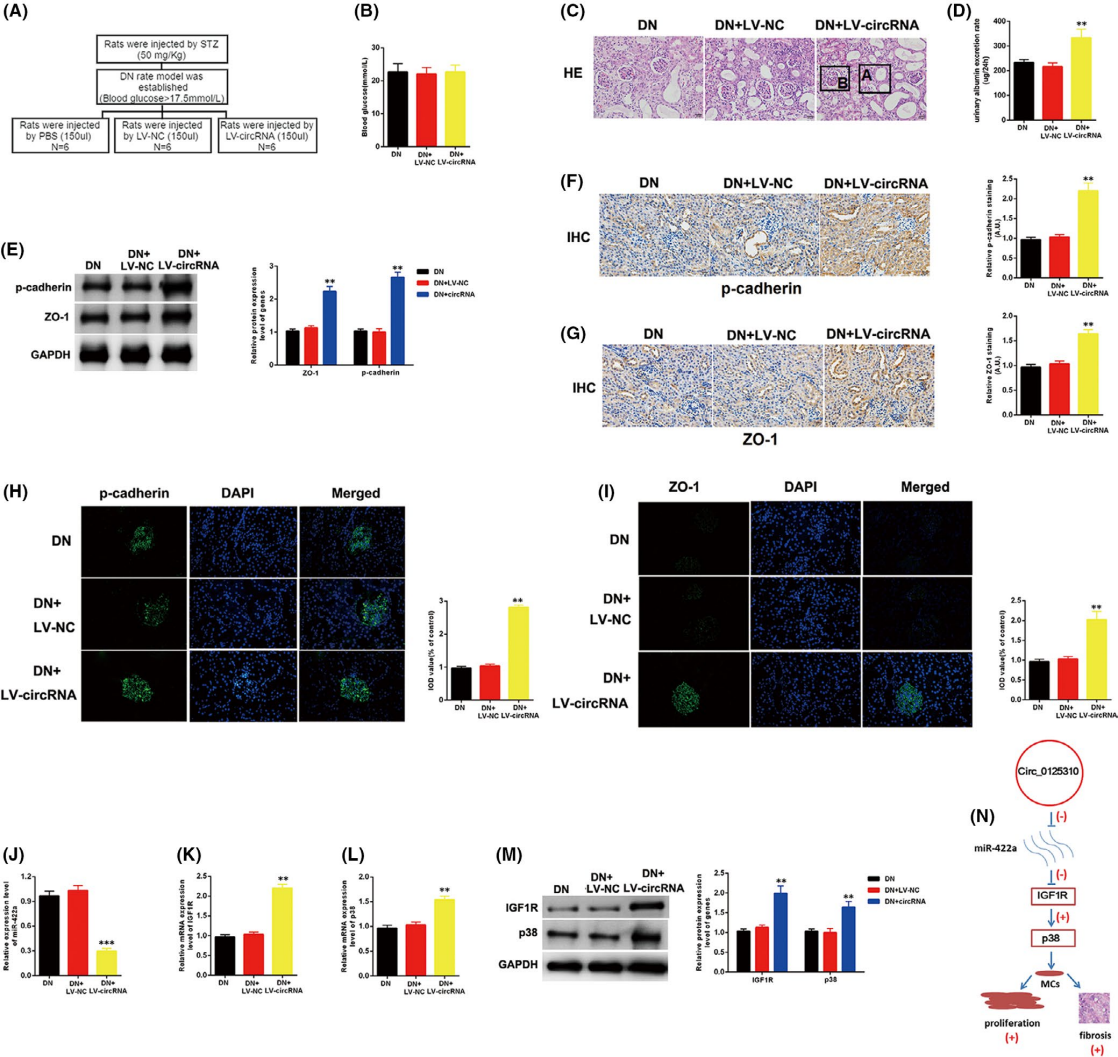
Overexpression of circ_0125310 promoted DN progression in vivo. A, Graphical representation of DN model treated with PBS, LV‐NC or LV‐circ_0125310. B, Blood glucose was detected in the DN+PBS group, DN+LV‐NC group and DN+LV‐circ_0125310 group. C, H&E staining results of the DN+PBS group, DN+LV‐NC group and DN+LV‐circ_0125310 group. Results indicated that the epithelium cells of tubule were more edematous in DN+LV‐circ_0125310 group. Then, glomerular mesangial proliferation was enhanced in DN+LV‐circ_0125310 group than DN+PBS group or DN+LV‐NC group. D, Urinary albumin excretion rate was measured in the DN+PBS group, DN+LV‐NC group and DN+LV‐circ_0125310 group. E, The expression level of p‐cadherin and ZO‐1 was measured in DN+PBS group, DN+LV‐NC group and DN+LV‐NC group. F, G, H, and I, The expression level of p‐cadherin and ZO‐1 was measured in DN+PBS group, DN+LV‐NC group and DN+LV‐circ_0125310 group tissues by IHC or IF assays. J, The expression level of miR‐422a was measured in DN+PBS group, DN+LV‐NC group and DN+LV‐NC group. K, L, and M, The expression levels of IGF1R/p38 axis were detected in DN+PBS group, DN+LV‐NC group and DN+LV‐NC group. N, Schematic diagram of mechanism on this research was showed

The graphical representation of the DN rat model treated with NC (PBS), LV‐NC and LV‐circ_0125310 is shown in Figure 7A. The blood glucose expression levels in the NC, LV‐NC and LV‐circ_0125310 groups were similar (Figure 7B). The H&E staining results showed that pathological changes, including swollen epithelium cells (A) and hyperproliferative glomerular MCs (B), were more numerous in the LV‐circ_0125310 group than those in the LV‐NC or NC group (Figure 7C). Moreover, the urinary albumin excretion rate was higher in the LV‐circ_0125310 group than that in the LV‐NC and NC groups (Figure 7D). Two fibrosis markers, namely, p‐cadherin and ZO‐1, were detected in the LV‐circ_0125310 group and the LV‐LV‐NC and NC groups. Western blot analysis results showed that the expression levels of p‐cadherin and ZO‐1 in the LV‐circ_0125310 group were higher than those in the LV‐NC or NC group (Figure 7E). As illustrated in (Figure 7F–I), IHC or IF assays showed that the expression levels of p‐cadherin and ZO‐1 in the LV‐circ_0125310 group were higher than that in the LV‐LV‐NC or NC group. Then, the miR‐422a/IGF1R/p38 axis was detected in the DN rat model. As shown in (Figure 7J–M), the expression level of miR‐422a in the LV‐circ_0125310 group was downregulated relative to that in the LV‐LV‐NC and NC groups. The expression levels of IGF1R and p38 in the LV‐circ_0125310 group were upregulated relative to those in the LV‐LV‐NC and NC groups. Finally, the schematic diagram of mechanism on this research is shown in Figure 7N. Overall, circ_0125310 promoted cell proliferation and fibrosis in DN via sponging miR‐422a and activating the IGF1R/p38 axis.

## 4 DISCUSSION

The poor control level of blood sugar is the major cause of the occurrence and development of DN.^29^ However, the concrete mechanism of action between high blood sugar and DN is largely unknown. Unsatisfactory blood glucose control is common among patients with DN treated with insulin.^30^ Thus, finding an effective therapeutic target is urgent.

Exosomes play an important role in diabetes complications. A recent study has illustrated that exosomes have become novel regulatory factors in diabetic heart, diabetes‐associated impaired wound healing, and diabetes‐induced cognitive disorders.^15^, ^31^, ^32^ However, the roles of exosomes in DN still require further characterization. Exosomes are separately isolated from MCs treated with high or normal glucose. The exosomes participate in the signal transduction between MCs and other cells.^33^ Our findings indicated that high glucose‐induced exosomes enhanced the growth and fibrosis in MCs. circRNAs were abundant in exosomes and participated in DN progression. A recent study has illustrated that circRNAs act as vital regulators in cell proliferation, fibrosis and inflammation in DN.^24^, ^25^ The qRT‐PCR results showed that the expression level of circ_0125310 was upregulated in high glucose‐induced exosomes. The in vivo experiments indicated that the effects of exosomes on MCs can be restored by silencing circ_0125310. circRNAs act as ceRNAs and participate in the development of DN.^23^ Our findings demonstrated that miR‐422a was the direct target of circ_0125310 through direct targeting and Ago2‐dependent manner. Then, the experimental results indicated that miR‐422a inhibited the proliferation and fibrosis of MCs by targeting IGF1R. The IGF1R plays a critical role in the development of DN. IGF1R regulates inflammation, cell growth and oxidative stress in the development of DN.^28^, ^34^, ^35^ p38 is the downstream signalling molecule of IGF1R. The IGF1R/p38 axis is an important phosphorylation activation signal of MAPKs.^36^, ^37^, ^38^ Previous studies have reported that the IGF1R/p38 axis participates in renal fibrosis.^28^, ^39^ Then, our findings indicated that high glucose‐induced exosomes upregulated the expression levels of IGF1R and p38. However, the effects of exosomes on the IGF1R/p38 axis can be restored by silencing circ_0125310. The ceRNA relationship between exo‐circ_0125310 and IGF1R was confirmed by dual‐luciferase reporter gene assays. Finally, in vitro experiments showed that the overexpression of circ_0125310 promoted the development of DN.

In conclusion, the exosome circ_0125310 secreted from MCs treated with high glucose promoted cell proliferation and fibrosis in DN via sponging miR‐422a and activating the IGF1R/p38 axis. This exo‐circ_0125310/miR‐422a/IGF1R/p38 network provided insight into DN progression and may contribute to the discovery of an effective therapeutic target for DN.

## CONFLICT OF INTEREST STATEMENT

The authors declare that there are no conflicts of interest.

## AUTHOR CONTRIBUTION

**Ying‐Chun Zhu:** Investigation (equal); Methodology (equal); Writing‐review & editing (equal). **Fangfang Zha:** Data curation (equal); Investigation (equal); Validation (equal). **Bo Tang:** Conceptualization (equal); Investigation (equal); Writing‐original draft (equal). **Ting‐Ting Ji:** Investigation (equal); Methodology (equal); Validation (equal). **Xiao‐Ying Li:** Data curation (equal); Validation (equal); Visualization (equal). **Linhong Feng:** Data curation (equal); Methodology (equal); Software (equal). **Shou‐Jun Bai:** Conceptualization (lead); Writing‐review & editing (lead).

## Data Availability

All data are available upon request. DR. SHOU‐JUN BAI (Orcid ID: 0000–0002–3717–5053).
